# Prediction of Axial Compressive Load–Strain Curves of Circular Concrete-Filled Steel Tube Columns Using Long Short-Term Memory Network

**DOI:** 10.3390/ma16093285

**Published:** 2023-04-22

**Authors:** Xinyu Fan, Fei Lyu, Jinglin Fan, Faxing Ding

**Affiliations:** 1 School of Automation, Central South University, Changsha 410075, China; 2 School of Civil Engineering, Central South University, Changsha 410075, China; 3 School of Architecture and Art, Central South University, Changsha 410075, China

**Keywords:** concrete-filled steel tubular stub columns, axial load–strain curve, machine learning, artificial neural network, long short-term memory network, machine-aided intelligent design

## Abstract

No study has been reported to use machine learning methods to predict the full-range test curves of circular CFST columns. In this paper, the long short-term memory (LSTM) network was introduced to calculate the axially compressive load–strain curves of the circular CFST columns according to an experiment database of limited scale. To improve the feasibility of input data for the recurrent neural network algorithm, data preprocessing methods and data configurations were discussed. The prediction results indicate that the LSTM network provides more accurate estimations compared with the artificial neural networks, random forest and support vector regression. Meanwhile, this method can be used to calculate the mechanical properties including the elastic modulus, ultimate bearing capacity, and the ductility of the columns with acceptable accuracy for engineering practice (the prediction error within 20%). For future research, it is expected that the machine learning method will be applied to predict the structural response of different members under various loading conditions.

## 1. Introduction

Concrete-filled steel tube (CFST) columns as structural components subjected to compression and bending have been recognized as a promising substitute for conventional reinforced concrete and steel columns in the construction of high-rise buildings and urban elevated girder bridges [1,2] amid the engineering community. The excellent mechanical performance of CFST mainly stems from the composite actions between the outer steel tube and the in-filled concrete, in which the steel tube provides the lateral confinement to the in-filled concrete, consequently reducing its lateral expansion behaviors. Meanwhile, the in-filled concrete prevents the inward local buckling of the steel tube, therefore upgrading the stability of the steel tube [3]. Such a beneficial mechanism has been experimentally and numerically examined in detail [4,5] and results in superior bending [6], axial bearing [7], and aseismic capacity [8] of the CFST columns in comparison with conventional structural columns. In the meantime, CFST columns emerged among other structural columns because of their economy and high resistance to specific loads as well. Concretely, using CFST columns can approximately reduce 60–70% usage of the concrete and 50% of the steel compared to the reinforced concrete and steel columns with the same bearing capacity, respectively. Moreover, the mutual protection of the steel tube and in-filled concrete gives CFST columns better fire [9], impact [10], and corrosion [11,12] resistances. Because of the above-mentioned advantages, composite structural columns based on similar concepts, such as recycled aggregate concrete-filled steel tube [13], concrete-filled aluminum alloy tube [14], lightweight concrete-filled steel tube [15], stirrup-confined concrete-filled steel tube [16,17], and concrete-filled dual steel tube columns [18], have been proposed in recent years to satisfy different design objectives for low carbon, good appearance, light weight, high strength, etc.

The axial compressive load–strain curve is one of the most fundamental and significant characteristics of structural columns in terms of safety design. Crucial design parameters including elastic modulus, ultimate bearing capacity, and ductility of the columns can be readily obtained once the load–strain curve is known. Consequently, the load–strain curve has always been the basic research objective of CFST columns during the past three decades. Generally, a theoretical derivation method making use of empirical parameters and finite element model analysis are the two major approaches to calculate the load–strain curves of CFST columns. Concerning the theoretical method-based studies, the stress–strain relations of the outer steel tube and the in-filled concrete are usually assumed initially, and the load–strain curve of the column is thereafter derived based on the equilibrium condition and predetermined interaction relationships in a stepwise manner. For instance, Susantha et al. [19] proposed the uniaxial stress–strain relation of concrete confined by differently shaped steel tubes in an earlier study. The proposed uniaxial constitutive relations were then applied to calculate the axial load–strain curves of CFST columns. However, the applicable scope of concrete strength in their study is 10–50 MPa. This indicates that the empirical parameter-based theoretical methods are inevitably limited to the material strength of the time. To expand the applications of the calculation method to high-strength materials, Sakino et al. [20] proposed stress–strain models for the in-filled concrete and the steel tube in CFST columns, respectively, based on a test database that is a part of the fifth U.S.–Japan Cooperative Earthquake Research Program. In particular, the scale effect on in-filled concrete was considered using a reduction factor. Similarly, Ding et al. [21], Choi and Xiao [22], Lai and Ho [23], Lin et al. [24], and Hu et al. [25], among others, proposed or modified the stress–strain relations of in-filled concrete and the steel tube to calculate the load–strain curves. It can be concluded from their results that although these models are derived based on the basic physical relationships, their applicability is gradually lower with the expansion of the scope of design parameters, because their assumed parameters in the stress–strain relationships were calibrated against fixed and limited experiment results.

Finite element model (FEM) analysis is another preferred approach to obtain the axial load–strain curves of CFST columns. In the FEM, the concrete and steel tube should be simulated by the appropriate element type, generally the solid element for concrete and the shell element for the steel tube. Meanwhile, the interactions between the surfaces of concrete and steel need to be defined using a contact model or spring model [4]. With the rational adoption of the constitutive relations and the adequate mesh, the load–strain curve of the CFST columns can be obtained. The FEM-based analysis of the axial loading behaviors of CFST columns was reported by Tao et al. [26], Han et al. [27] and Nguyen et al. [28], among others. These studies revealed that the FEM method is effective for analyzing the composite action of CFST columns, since the stress and strain of concrete can be calculated but is hard to measure in the experiment (there is a method to measure the force absorbed by concrete in CFST columns [25]). However, the limitation arises as well because the passive confinement effect cannot be reflected in the model. To consider the load path effect in the axially loaded CFST columns, analytical models [24,29] were proposed and applied to the fiber element-based modeling of CFST columns. This method, however, requires a lot of assumptions and complicated iterations for determining the confinement path. For the FEM method, because of the great number of parameters involved in the different analyzed constitutive laws, it is very difficult to predict the effects both in terms of ultimate strength and/or ultimate strain of the different assumptions and calibration option, as well [30].

Civil engineering has recently witnessed the remarkable development and widespread application of soft computing methods and intelligence technology [31,32]. However, the applications of machine learning algorithms in CFST research are still in initial and exploratory stages. Most existing studies focus on the prediction of the mechanical parameters (e.g., axial bearing capacity and stiffness) based on the collected experimental database [33]. Different algorithms, including Artificial Neural Network [34,35,36,37], Gene Expression Programming [38,39], Support Vector Regression (SVR) [33], and the Adaptive Neuro-Fuzzy Inference System [40], were reported to be accurate and efficient in predicting the capacities of CFST members. For predicting continuous responses of loaded members, there are a few practices, such as using an AI-based cognitive framework for evaluating the thermal and structural response of concrete structures under elevated temperatures [41]. However, to the authors’ knowledge, there is only one study that has reported on using the machine learning method to calculate the complete load–strain curves of CFST members [42]. Moreover, in that study [42], the load–strain curves of CFST columns are produced by connecting specific points predicted by an ANN-based model, namely, not predicting continuous structural responses. Currently, the metamodel [43] (or surrogate model) is becoming a promising alternative to the conventional computing approaches in the civil and material engineering fields. Hence, it is essential to expand the application scope of the machine learning method from predicting several mechanical properties to predicting the full-range performance of structural components subjected to different loading conditions. However, a large obstacle to implementing the machine learning methods in structural engineering problems is the small scale of the experiment database. Therefore, for the prediction of the full-range performance of structural members, effective utilization of a limited database is of greater importance than the aforementioned single-valued prediction problems. Hence, in the present study, the processing of tested curve data was discussed in detail. Different input data configurations were compared so that the crucial curve features could be captured. Meanwhile, although some widely used machine learning methods (e.g., ANN and SVR) are applicable in predicting the mechanical properties which are in one-dimensional form, their accuracy in predicting the load–strain curve (which are in two-dimensional form) is questionable. To solve this problem, Long Short-Term Memory Network (LSTM) [44], a method that is particularly effective in dealing with time series data, was applied in this study.

This paper is organized as follows: Section 1 introduces the background and motivation; the load–strain curve of CCFST columns and its characteristics are illustrated in Section 2; Section 3 describes the constructed experimental database and the processing methods of collected data; the adopted algorithms and the implementation are explained in Section 4; Section 5 presents the results of prediction using the LSTM network as well as other comparison methods. Section 6 ends the paper with major conclusions.

## 2. Axial Load–Strain Curve of CCFST Columns

**Figure 1:**
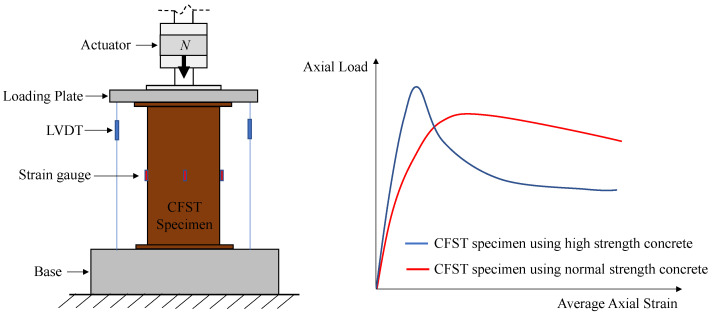
Schematic diagram of compressive test of CCFST columns and typical measured axial load–strain curves.

The axial load–strain curve data of CCFST columns of interest in this paper are those obtained by the compressive test. As shown in Figure 1, the column specimen is concentrically compressed by an actuator and a load plate so that the steel tube and in-filled concrete are simultaneously loaded. The relative displacement between the load plate and the apparatus base is measured by several Linear Variable Differential Transformers (LVDTs) and divided by the length of the specimen to obtain the average axial strain, while the axial load is measured by directly reading the reactional force of the actuator. The right half of Figure 1 illustrates the typical shapes of the axial load–strain curves of CCFST columns. Generally, the scale of the curves varies a lot according to the change of design parameters; however, the shape of the curves can be roughly categorized into two types, as shown in the figure. For most CCFST columns with conventional materials and dimensions, the stress–strain curves are similar in shape to the red line in Figure 1. For such a type, the in-filled concrete is generally well-confined during the whole loading process and no sudden drop in their bearing capacity occurred. On the contrary, for CCFST columns using high-strength concrete or with a thin-walled steel tube, the stress–strain curves might be similar in shape to the blue line in Figure 1. In this situation, a sudden drop in the axial load occurs after the peak point. Such a transition is ascribed to the brittleness of the high-strength concrete and the local buckling of the thin-walled steel tube. Hence, the anticipated difficulties of using machine learning methods in predicting the stress–strain curves of CCFST columns are to distinguish between two shapes of curves and to determine the scale of the curves according to the input design parameters.

## 3. Construction and Processing of the Database

Many studies have been reported to utilize machine learning methods to calculate the ultimate bearing capacity of CFST columns. For circular columns, the number of the collected experiment specimens is generally around 400 to 1000. However, many of those studies only reported the value of bearing capacity without giving the complete axial load–strain curves, which results in a smaller database of trials and higher predictive difficulty in this study. In this paper, the available axial load–strain curves of circular CFST columns in the literature were collected to construct a database. As shown in Table 1, 104 curve datasets from 10 studies were collected, covering a relatively wide range of design parameters. Generally speaking, the constructed database covered the commonly used material strength and dimensions both in engineering practice and academic research. In this study, five basic design parameters, including diameter of the column *D*, tube wall thickness $t_{s}$, column height *H*, yield strength of steel tube $f_{s}$, and compressive strength of in-filled concrete $f_{c}$ are used for predicting the compressive behaviors of the CCFST columns.

After data collection is complete, it is necessary to process and regularize them for a better adaptability to the algorithm. By analyzing the collected data, it can be found that when the tested axial load–strain curve has an obvious peak point, the corresponding axial strain of the peak load point is generally smaller than 10,000 με.

**Figure 2:**
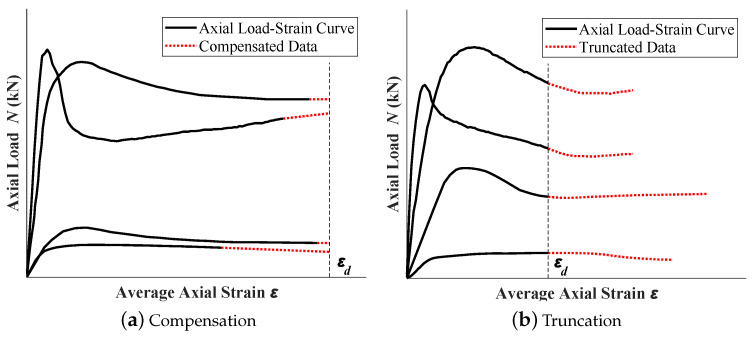
Preprocessing of collected experiment data.

Meanwhile, if the tested curve does not have an obvious peak point, most researchers and design codes define the axial load corresponding to 10,000 με as the bearing capacity of the specimen, since the allowable deformation in practical engineering is far less than this value. The collected experimental curves vary greatly in their measured maximum axial strain; however, the effective information (elastic modulus, ultimate bearing capacity, and ductility) are contained by the earlier part of the curve. As a result, in this study, a limit of axial strain of 25,000 με was set to ensure the peak load point and the post-peak behaviors were captured. Based on this principle, the collected axial load–strain curve data were processed, as shown in Figure 2. As shown in Figure 2a, if the measured test data are short, a compensation operation will be performed and will extend the curve to the set limit (red dashed line in the figure). The compensation is a straight line with the slope of the final phase of the measured curve. If the measured curve is long, a truncation operation will be performed to discard the data after the set limit, as shown in Figure 2b, while the effective information was retained. After the aforementioned data preprocessing, the collected axial load–strain curve data have the unified size.

## 4. Methodology

### 4.1. Long Short-Term Memory Network

**Figure 3:**
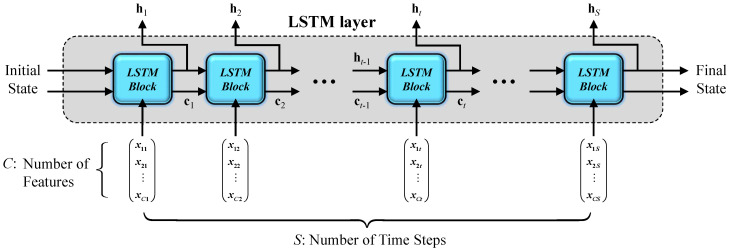
LSTM layer.

**Figure 4:**
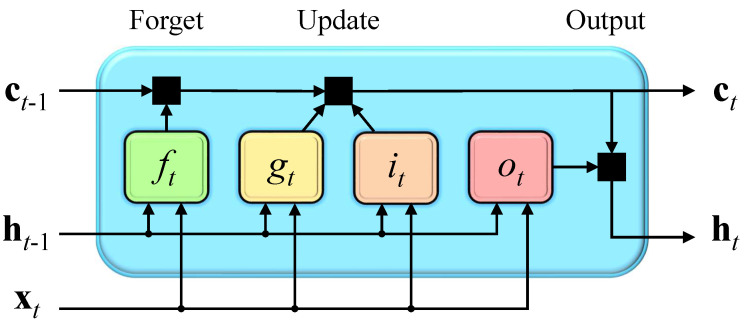
LSTM block.

Unlike previous studies which use the machine learning method to predict a single value (e.g., axial or flexural bearing capacity), each data sample in this curve prediction problem is a two-dimensional data series. Therefore, it is questionable that those commonly used algorithms (ANN, SVR, and RF, among others) can be successfully applied to the present study as reported in those past single-value prediction works. On the other hand, with the emerging and rapid development of machine learning technology, recurrent neural networks (RNNs) were proposed specifically to process time series data [54]. The main difference between RNNs and traditional feed-forward networks is the internal states, which can analyze series information and learn time features [55]. Moreover, Hochreiter and Schmidhuber [44] proposed the LSTM, which is a special variant of RNN structure, to handle long time series and overcome the vanishing and exploding gradients in the training process of RNNs. In this study, the axial load of the columns with regard to the axial strain can be treated as a time series, since the axial strain increases monotonously with time. Hence, its capacity in predicting complete axial load–strain curves of CCFST columns can be expected. Without loss of generality, a time series *X* with *C* features of length *S* through an LSTM layer is shown as Figure 3 [56]:

where $h_{t}$ is the output (also known as the hidden state) and $c_{t}$ is the memory cell state at time step *t*, respectively. At time step 1, the LSTM block obtains the first hidden state $h_{1}$ and the first memory cell state $c_{1}$ by using the initial state and the first time step of the series $x_{1}$. At time step *t*, the LSTM block obtains current hidden state $h_{t}$ and the updated memory cell state $c_{t}$ by using the last state $\left(c_{t-1},h_{t-1}\right)$ and the current time step of the series $x_{t}$. At each time step, the LSTM block adds information to or removes information from the memory cell state. These updates are controlled by the LSTM block composed of the input gate $i_{t}$, the output gate $o_{t}$, the forget gate $f_{t}$, and cell candidate $g_{t}$ at time step *t*, as shown in Figure 4 [57].

As shown in Figure 4, the forget gate first controls how much historical information is stored and removed. Then, the input gate decides whether to allow the input layer information to enter the current memory cell. Finally, the output gate determines the final output of the block. The update equations of LSTM were applied to implement the above processes. For current input $x_{t}$ and hidden state $h_{t}$ at time step *t*, the operations can be expressed as follows:

(1) $$\begin{array}{cc} \; & i_{t}=\sigma \left(W_{i}x_{t}+R_{i}h_{t-1}+b_{i}\right)\\ \; & f_{t}=\sigma \left(W_{f}x_{t}+R_{f}h_{t-1}+b_{f}\right)\\ \; & g_{t}=\sigma \left(W_{g}x_{t}+R_{g}h_{t-1}+b_{g}\right)\\ \; & c_{t}=f_{t}⊙\left(c_{t-1}+i_{t}⊙g_{t}\right)\\ \; & o_{t}=\sigma \left(W_{o}x_{t}+R_{o}h_{t-1}+b_{o}\right)\\ \; & h_{t}=o_{t}⊙\tanh\left(c_{t}\right) \end{array}$$

where the learnable weights $W_{i}$, $W_{f}$, $W_{g}$, $W_{o}$ are the input weights, $R_{i}$, $R_{f}$, $R_{g}$, $R_{o}$ are the recurrent weights, and $b_{i}$, $b_{f}$, $b_{g}$, $b_{o}$ are the gate bias vectors for the input gate, the forget gate, the cell candidate and the output gate, respectively. The σ(·) represents the sigmoid activation function that $\sigma \left(x\right)={\left(1+e^{-x}\right)}^{-1}$. The tanh is the hyperbolic tangent activation function and the ⊙ is the Hadamard product (element-wise multiplication of vectors).

### 4.2. Curve Prediction Data Configurations

The axial load–strain curve can be approximately formulated as the following non-linear equations:

(2) $$N=f_{\theta }\left(\varepsilon \right)$$

where ε is strain, *N* is axial load, and $\theta =(D,t_{s},H,f_{s},f_{c})$ is the vector of five design parameters. In order to solve the axial load–strain curve prediction problem, the curve is discretized into *S* points, i.e., the ε and *N* are discretized into series $\varepsilon =(\varepsilon_{1},\varepsilon_{2},...,\varepsilon_{S})$ and $N=(N_{1},N_{2},...,N_{S})$, where the number of discrete points and the series length *S* correspond to the number of time steps in LSTM. The points in the axial load–strain curve can be regarded as the time series in LSTM for the curve prediction. The basic curve prediction problem is modeled as the problem of predicting the relationship of strain series ε and axial load series N with five design parameters θ.

As mentioned in Section 3, the strain ε has unified size after compensation and truncation. As long as all curves are discretized into the same number of points, the strain series ε of all curves are the same fixed series. At this time, the relationship between strain and axial load can be obtained by taking the strain series ε and five design parameters θ as the input of the prediction algorithm and the axial load series N as the output of the prediction algorithm.

**Figure 5:**
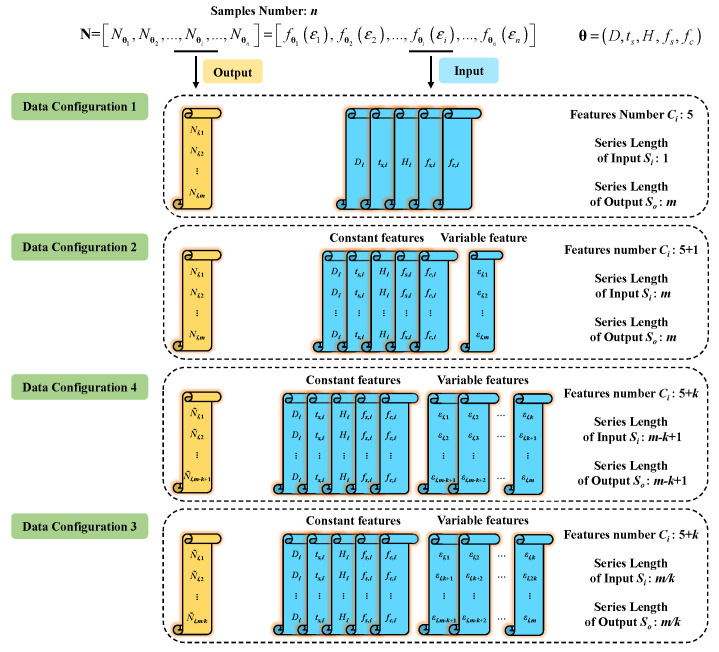
Data configurations.

The curve prediction problem can be constructed by different input configurations $\in R^{n\times C_{i}\times S_{i}}$ and output data configurations $\in R^{n\times 1\times S_{o}}$, where *n* is the samples number, $C_{i}$ is the number of input feature, $S_{i}$ is the series length of input, and $S_{o}$ is the series length of output. Four data configurations are used, as shown in Figure 5, to obtain better curve prediction accuracy. In order to represent the difference in series length *S* in different configurations, we use the symbol *m* to represent the series length of output $S_{o}$ in configuration 1 and the series length $S_{i}$ and $S_{o}$ in configuration 2.

**Figure 6:**
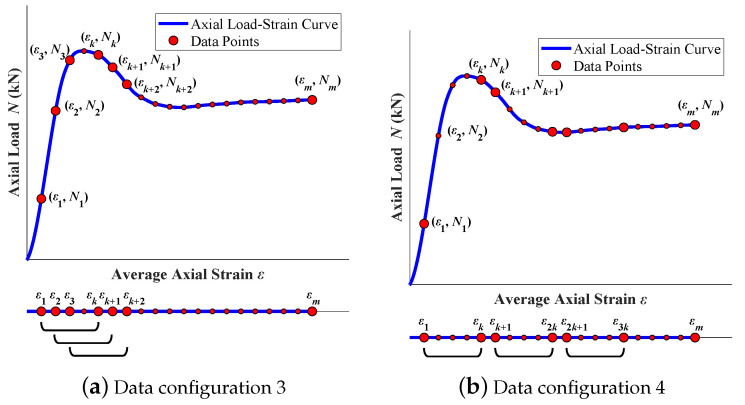
Data configuration.

(1) In data configuration 1, the five design parameters θ are set as input $\in R^{n\times 5\times 1}$, and the axial load series N is set as output $\in R^{n\times 1\times m}$, regardless of the strain series ε. Since only five design parameters θ are inputs to the prediction algorithm, the number of the input feature is $C_{i}=5$ and the series length of the input is $S_{i}=1$. The outputs of the prediction algorithm are the axial load series N, so the series length of the output is $S_{o}=m$. This configuration can be used for curve prediction by the ANN method.

(2) In data configuration 2, the design parameters θ and the strain series ε are set as input $\in R^{n\times (5+1)\times 1}$, and the axial load series N is set as output $\in R^{n\times 1\times m}$. The five design parameters θ are five constant features, while the strain series ε are variable features. Therefore, the total number of input features is $C_{i}=5+1$. The length of the strain series *m* is taken as the series length of the input $S_{i}=m$, while the length of the output axial load series *m* is taken as the series length of the output $S_{o}=m$. In this configuration, each point on the axial load–strain curve is regarded as one row in the series, including the design parameters θ of the sample and its strain value ε. This configuration can be used for curve prediction both by some basic machine learning methods after flattening and LSTM.

(3) In data configuration 3, the design parameters θ and the strain series ε are set as input $\in R^{n\times (5+k)\times (m-k+1)}$, and the series N is set as output $\in R^{n\times 1\times (m-k+1)}$. Each row of data configuration 2 has five constant features and only one variable feature. As a result, the number of variable features is much lower than that of constant features. Data configuration 3 uses sliding windows to expand variable features, resulting in a higher number of input variable features than data configuration 2, as shown in Figure 6a. The number of strain points ε in a sliding window, which is defined as *k*, is the number of variable features. Each sliding window of strain points forms a row in the input series with five design parameters θ. Therefore, the total number of input features is $C_{i}=5+k$. After variable features are expanded by sliding windows, the length of the input is reduced to $S_{i}=m-k+1$. Accordingly, the length of the axial load output $S_{o}=m-k+1$. The axial load value of a sliding window $\tilde{N}$ is obtained by calculating the average axial load value *N* corresponding to all strain points in this sliding window.

(4) In data configuration 4, the design parameters θ and the strain series ε are set as input $\in R^{n\times (5+k)\times (m/k)}$, and the series N is set as output $\in R^{n\times 1\times (m/k)}$. Different from data configuration 2, data configuration 4 is built for variable feature expansion using uncrossed segments, as shown in Figure 6b. All strain points are divided into equal uncrossed segments in order. The number of strain points in a segment is defined as *k*. Each segment of strain points forms a row in the input series with five design parameters θ. Therefore, the total number of feature inputs $C_{i}=5+k$. After variable feature expansion by uncrossed segments, the length of the input is reduced to $S_{i}=m/k$. Accordingly, the length of the axial load output $S_{o}=m/k$. The axial load value of a sliding window $\tilde{N}$ is obtained by calculating the average axial load value *N* corresponding to all strain points in this segment.

### 4.3. LSTM Network Structure

As the prediction target, the axial load–strain curve is smooth and continuous. In order to realize curve prediction, curve data are discretized into a series of points. Considering the relationship between adjacent points instead of taking each point as an independent data point can better predict and restore the axial load–strain curve. LSTM is suitable for processing time series and series data as it can learn long-term dependencies between points in time series data. Using LSTM to predict the axial load–strain curve can restore a smooth curve relatively smoothly and accurately. The LSTM network structure used in this paper is composed of the following layers in order: Input Layer, LSTM Layer, Fully Connected Layer, Dropout Layer, Fully Connected Layer, Regression Layer.

**Figure 7:**
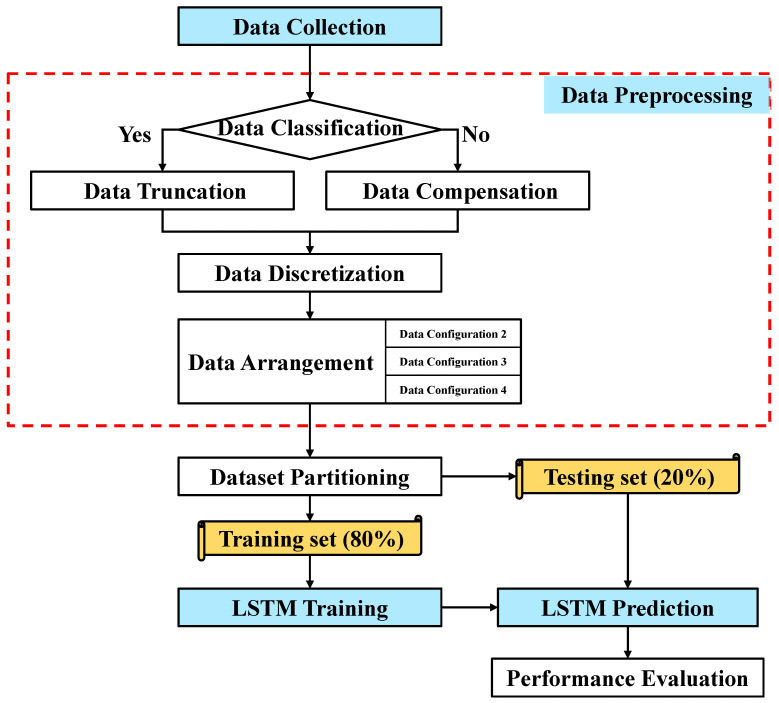
Flowchart of proposed method.

The proposed flowchart of the axial load–strain curve prediction method is shown in Figure 7. It can be divided into four steps: (1) Data Collection. The data used in this paper are collected from 10 papers. (2) Data preprocessing. This step mainly includes Data Truncation, Data Compensation, Data Equidistant Discretization, and Data Arrangement. (3) LSTM training. The LSTM network uses a training set to train the prediction model. (4) LSTM prediction. The LSTM network uses a testing set to predict the axial load–strain curve and conduct performance evaluation.

## 5. Prediction of Axial Load–Strain Curve of CCFT Columns Using LSTM Network

### 5.1. Comparison of the Accuracy of Different Algorithms and the Applicability of Data Configurations

The prediction of axial load–strain curves of CCFST columns was performed by using LSTM, ANN, RF, and SVR algorithms with different data configurations. For comparison, a fixed training set and test set were selected from the aforementioned experimental database, among which were 84 curve datasets for training and 20 curve datasets for testing. The detailed information of selected specimens is shown in Table 2. In this paper, the root mean square error (RMSE) between the predicted and test results is adopted to evaluate the performance of the algorithm. The RMSE of each load–strain curve can be expressed as

(3) $$RMSE=\sqrt{\frac{1}{n}\sum_{i=1}^{n}{\left(test_{i}-predicted_{i}\right)}^{2}}$$

where *n* is the number of discrete points of the axial load–strain curve. In this study, *n* is defined as a constant of 100, while the performance of the algorithm on the whole training/test set is assessed by the average RMSE value of 1000 repeated predictions of all curve data. In the following discussion, the performance of the algorithm is presented in the order of the data configurations.

**Figure 8:**
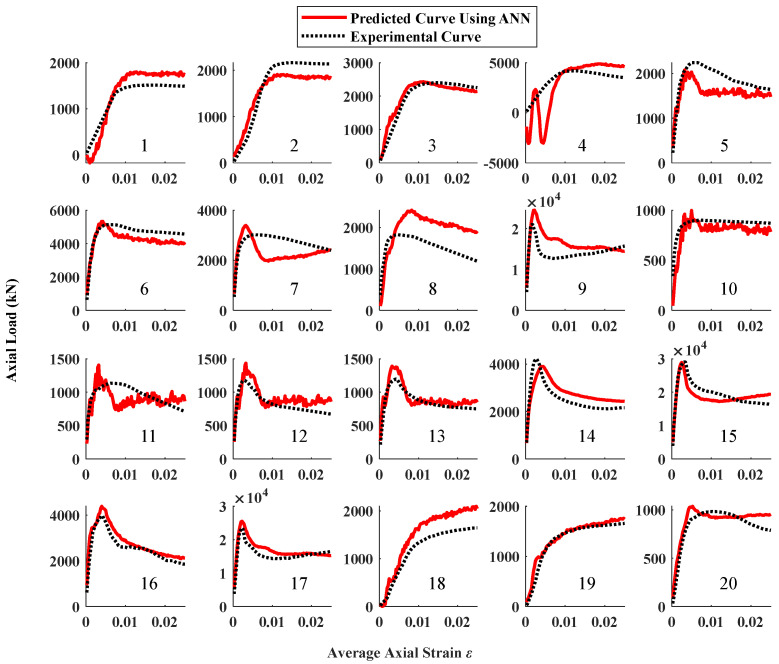
Comparison between experimental results and predicted results using ANN and data configuration 1 of test set.

In data configuration 1, the input parameters are only the 5 design parameters (*D*, $t_{s}$, *H*, $f_{y}$, and $f_{c}$), while the required outcome is the completed axial load–strain curve (as shown in Figure 5). Only the ANN can deal with this data configuration without additional modifications to the algorithm structure. The average RMSE of the ANN of 1000 repeated predictions on the whole test set is 920 kN (as shown in Table 3), which is the highest among all comparative items. The comparison between the predicted curves of 1 time (random) and the experimental curves are shown in Figure 8. It can be observed from the figure that the predicted curves are coarse with many fluctuations. Meanwhile, the results of some specimens remarkably deviated from the test value (No. 4, 7, 8, 11, and 12 in Figure 8). These results indicate that using only the design parameters as the input is insufficient for the machine learning method to predict a completed axial load–strain curve for the CCFST column. Moreover, the ANN algorithm is unstable in this case.

**Figure 9:**
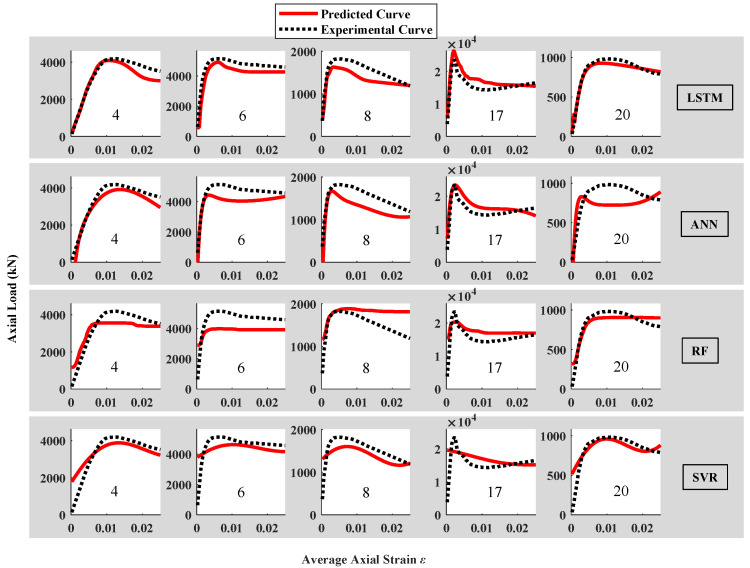
Comparison between experimental results and predicted results using different algorithms and data configuration 2 of the test set.

In addition to 5 design parameters, the strain series is added as the input in data configuration 2 (as shown in Figure 5). The motivation is to ensure the algorithm developing a better understanding of the form of the predicted target. For the LSTM algorithm, this data configuration can be directly applied. For ANN, RF, and SVR algorithms, an additional flatten layer is required. The results of 4 algorithms using data configuration 2 are shown in Table 3 and Figure 9. The average RMSE values of 1000 repeated predictions are 557, 735, 786, and 723 kN, respectively. It can be seen that the LSTM provided the most accurate and stable results among other comparison algorithms. Moreover, the different curve types (No. 17 and 20) can be appropriately reproduced. Figure 9 reveals that it is of great importance to add the strain series as the input for the curve prediction, since the results of ANN improved significantly and noticeably smoothed compared with data configuration 1.

**Figure 10:**
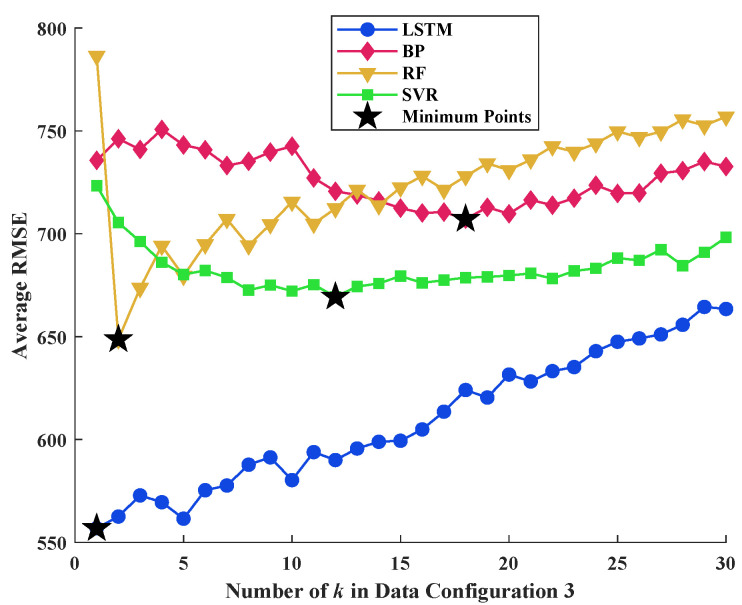
Comparison of average RMSE of 4 algorithms with different *k* for data configuration 3.

**Figure 11:**
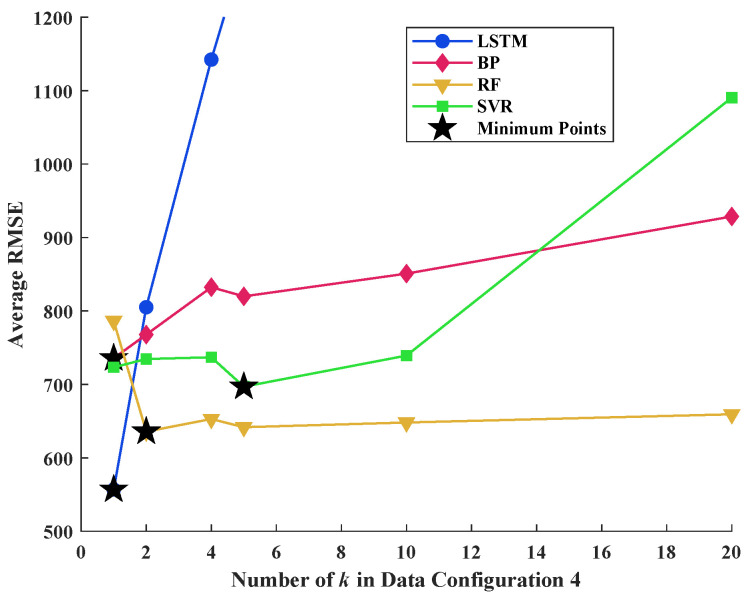
Comparison of average RMSE of 4 algorithms with different *k* for data configuration 4.

Data configurations 3 and 4 can be regarded as the different varieties of data configuration 2. In data configuration 3, the idea of the sliding window algorithm is adopted. It is expected that such an operation can enhance the understanding of algorithms in the context of the data series, while in data configuration 4, the data series is divided into uncrossed segments so that the algorithm may pay more attention to the local characteristics of the curves. The average RMSE values of 1000 repeated predictions of 4 algorithms with different *k* (window length for data configuration 3 and segment length for data configuration 4) are plotted in Figure 10 and Figure 11.

According to the definition of data configuration, when *k* equals to 1, data configurations 3 and 4 degenerate into data configuration 2. Based on Figure 10, the LSTM provides the lowest average RMSE among the 4 algorithms. However, the performance of the LSTM algorithm became worse with the increase in the sliding window length *k*. Similar trends were observed from Figure 11, indicating that the processing of input data form cannot enhance the accuracy and stability of the LSTM algorithm in this study. Although there are minor improvements on the performance of ANN, RF, and SVR with the variation in *k*, their performance is generally inferior to the LSTM algorithm.

Based on the above discussion, questions raised in the introduction section can be answered. Although the data volume in structural engineering problems is small, it is feasible to conduct a prediction of mechanical behaviors with machine learning methods if the proper algorithm is adopted. Compared with ANN, RF, and SVR algorithms, the LSTM algorithm has a better adaptability to handle the two-dimensional prediction problems (curve prediction problem). The attempts to improve the data configurations have not produced positive results. This may be ascribed to the small data size and length. However, it is still worth trying if the prediction target is more complex with larger data sets.

### 5.2. Comparison of the Accuracy of LSTM Algorithms and Finite Element Model Analysis

To illustrate the application prospect of the proposed method, it is necessary to compare its performance with the most commonly used method, the finite element model analysis, in structural analysis. To this end, the finite element modeling approach of CCFST columns proposed by xing Ding et al. [58] using ABAQUS was implemented here to calculate the axial load–strain curves of the 20 specimens in the test set. To save space, the detailed information of the modeling method including the element types, constitutive relations, interactions, loading methods, and boundary conditions are not explained in this paper and can be referred to in [58]. It should be mentioned here that the adopted method has been verified against a large amount of test results and has been applied to investigate the mechanical behaviors of CCFST columns under axial and eccentric compression [59], bending, torsion [60], and cyclic loading [61] in previous studies. Hence, it is rational to use this model herein to evaluate the proposed machine learning method.

**Figure 12:**
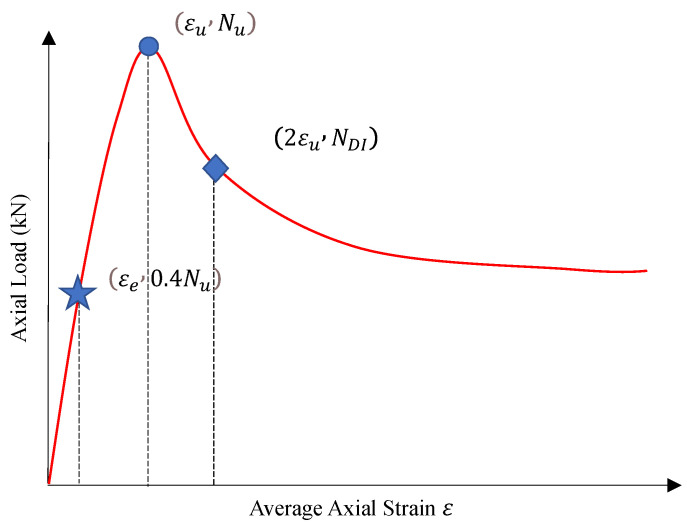
Definition of the key information points on axial load–strain curves of CCFST columns.

As mentioned in the introduction, the axial load–strain curve of CCFST columns is valuable because it provides vital information for the structural member design. In this study, we defined three key information points on the axial load–strain curves of CCFST columns as shown in Figure 12. In the figure, $\varepsilon_{e}$ and $\varepsilon_{u}$ are the strains corresponding to the points to calculate the elastic modulus *E* and ultimate strength $N_{u}$. Meanwhile, in order to quantitatively describe the deformation capacity of the specimens, a point where the x-coordinate is 2 times $\varepsilon_{u}$ is defined. In the following discussion, the performance of the LSTM algorithm and finite element model analysis is compared on their accuracy on these important points.

**Figure 13:**
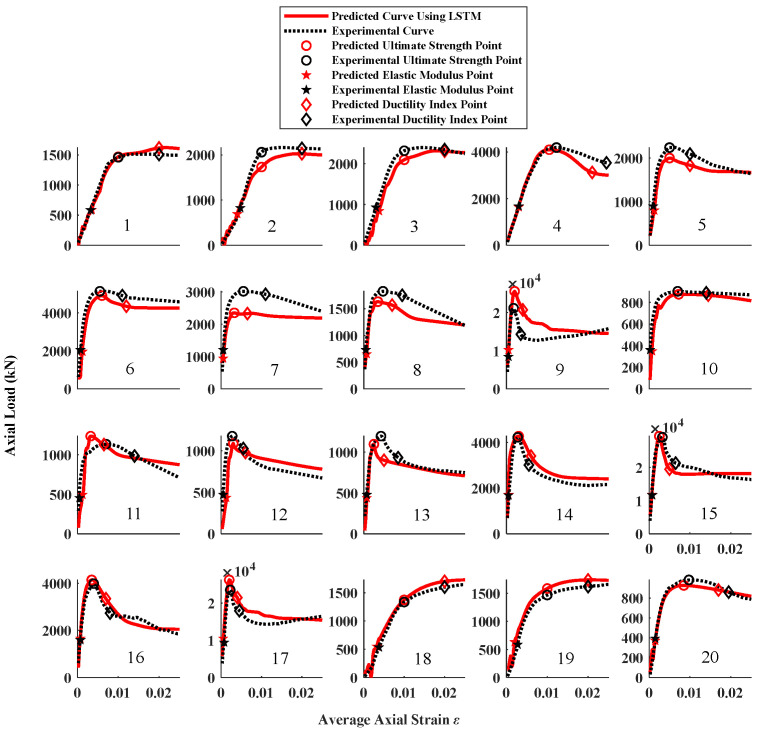
Comparison between experimental results and predicted results using LSTM algorithm.

**Figure 14:**
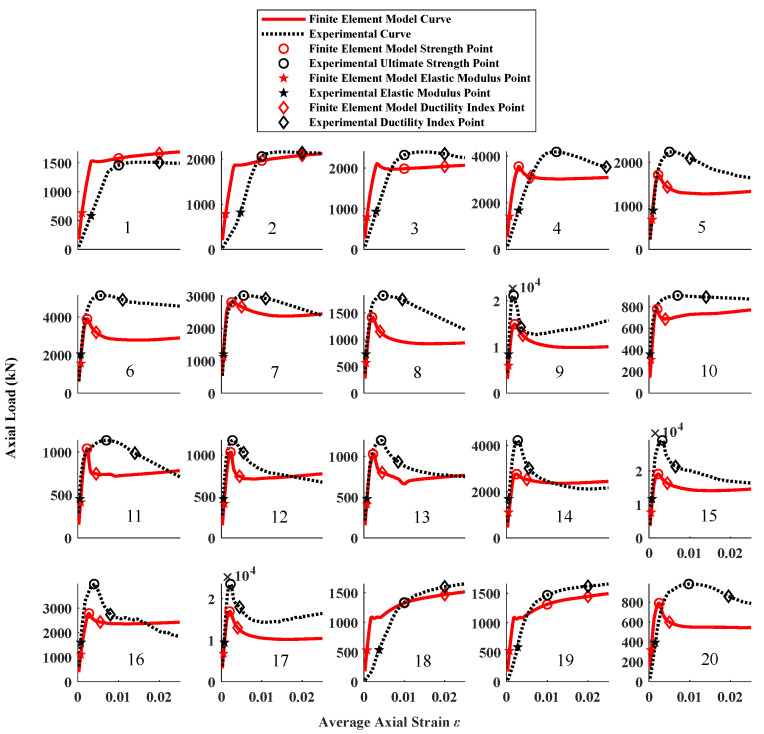
Comparison between experimental results and predicted results using finite element model analysis.

The complete results of the LSTM algorithm and finite element model analysis on the test set are shown in Figure 13 and Figure 14. It can be observed from the figure that the overall performance of the LSTM algorithm is evidently better than the adopted finite element modeling approach. However, we cannot easily assume that the machine learning method is more accurate than the finite element model analysis. The advantage of the machine learning method is that it can consider intricate factors in the real world. Such intricate factors include test methods, instrument errors, and even errors. In this study, the experiment database is obtained by collecting data from 10 studies. We can also assume that data from the same study have similar characteristics (i.e., the precision of the test and the quality of materials). These data are then broken up into the test set and training set. As a result, the LSTM algorithm inherits these factors from the training set and reflects them in the test set. Hence, it can be summarized that the LSTM algorithm has a better adaptability to solve practical problems in structural engineering.

**Figure 15:**
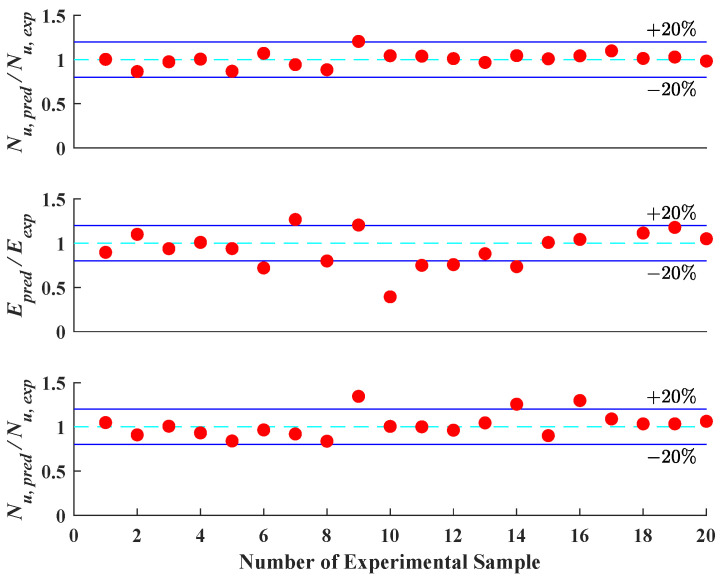
Prediction accuracy of key points using LSTM algorithm.

**Figure 16:**
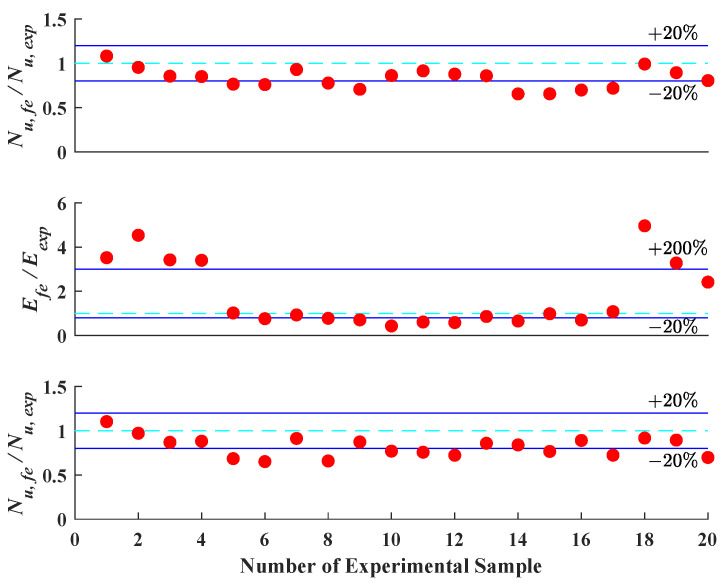
Prediction accuracy of key points using finite element model analysis.

In Figure 15 and Figure 16, the prediction accuracy of the LSTM algorithm and finite element model analysis is presented. As revealed by the figures, the LSTM algorithm provides comparable prediction accuracy on the vital information of the axial load–strain curve of CCFST columns with the finite element model analysis. The prediction error for stiffness, ultimate strength, and deformation capacity defined in this paper is generally within 20%. This indicates that the LSTM algorithm has a sound application prospect for structural design.

## 6. Conclusions

Machine learning algorithms have been flourishing in various fields in recent years. For analyzing the structural performance, using machine learning methods inevitably confronts the problem of few-shot learning. It is useful to select suitable algorithms and data processing for different problems. In this study, the application of a machine learning algorithm is extended to predict the complete load–strain curves of a structural member under a specific load. Based on the observations made in this paper, the LSTM algorithm is proved to be effective in curve prediction problems. For the problem studied in this paper, the LSTM algorithm provides comparable accuracy to the commonly used finite element model analysis in calculating the axial load–strain curves of CCFST columns. It is expected that the proposed method can enhance the efficiency and precision of the design of CCFST columns. In future work, we will use load–strain curve data and ultimate bearing capacity data simultaneously as different forms of data to train machine learning models, aiming to improve prediction accuracy. Moreover, we will also employ machine learning methods to investigate the behavior of CFST columns under eccentric compression.

## Figures and Tables

**Table 1:** Axial load–strain curve data of CCFST columns in the literature.

Author	No.	*D* (mm)	$t_{s}$ (mm)	*H* (mm)	$f_{s}$ (MPa)	$f_{c}$ (MPa)
Zhou et al. [45]	12	140.84–262	2.11–3.94	525–975	691, 734	42.4, 45.4
Zhang [46]	11	153–474	1.54, 9.83	306–948	290–345	77.1
Zhu et al. [47]	3	557.65–559.40	16.52–16.54	994.85–997.50	546	31.7
Ekmekyapar and Al-Eliwi [48]	18	114.30	2.74–5.90	300, 600, 900	235–355	56.2–107.2
Wang et al. [49]	12	215.9–632.1	2.6–11.2	657–1890	259.8–590.4	49.64
de Oliveira et al. [50]	16	114.30	3.35	342.9–1143.0	287.33	32.7–105.5
Wang et al. [51]	4	215.4–817.4	2.5-9.0	657–2460	276.0–590.4	41.24
Xiong et al. [52]	16	114.3, 219.1	3.6–10.0	250, 600	300–428	51.6–193.3
Chen et al. [29]	9	111.0–187.1	1.7–5.5	333.0–561.3	248.3–354.0	46.2–95.0
Huang et al. [53]	3	200–300	2.0–5.0	840	265.8–345.7	27.2–31.2
Total	104	111.0–817.4	1.7–16.54	250–2460	235.0–734.0	27.2–193.3

**Table 2:** Design parameters of specimens in test set.

Number	*D* (mm)	$t_{s}$ (mm)	*H* (mm)	$f_{s}$ (MPa)	$f_{c}$ (MPa)
1	140.84	2.37	525	734	42.4
2	151.05	2.98	562.5	691	42.4
3	181.8	2.17	675	734	45.4
4	262	2.16	975	734	45.4
5	153	3.64	306	322	77.1
6	235	5.66	470	290	77.1
7	280	4.0	840	272.6	31.2
8	182.7	2.01	548.1	313	46.2
9	628.5	6.9	1890	276	41.24
10	114.3	3.35	571.5	287.3	58.7
11	114.3	3.35	342.9	287.3	88.8
12	114.3	3.35	571.5	287.3	88.8
13	114.3	3.35	800.1	287.3	88.8
14	220.7	4.3	657	325.1	49.64
15	626	11.2	1890	269.1	49.64
16	215.9	2.6	657	590.4	49.64
17	625.6	7.2	1890	276	49.64
18	114.3	5.9	300	355	56.2
19	114.3	5.9	600	355	56.2
20	114.3	2.74	900	235	66.75

**Table 3:** Performance of different algorithms using different data configurations.

Average RMSE * of	Configuration 1 (kN)	Configuration 2 (kN)	Configuration 3 (kN)	Configuration 4 (kN)
LSTM	-	557	557	557
BP	920	735	707	735
RF	-	786	649	635
SVR	-	723	669	694

* average value of 1000 repeated predictions.

## Data Availability

Some or all data will be available upon request from the corresponding author.
